# Factors affecting driving performance in patients with Multiple Sclerosis – still an open question

**DOI:** 10.3389/fneur.2024.1369143

**Published:** 2024-02-28

**Authors:** Susan Seddiq Zai, Roshan das Nair, Christoph Heesen, Carsten Buhmann, Anya Pedersen, Jana Pöttgen

**Affiliations:** ^1^Institute of Neuroimmunology and Multiple Sclerosis (INIMS), Center for Molecular Neurobiology, University Medical Center Hamburg-Eppendorf, Hamburg, Germany; ^2^Health Division, SINTEF, Trondheim, Norway; ^3^School of Medicine, University of Nottingham, Nottingham, United Kingdom; ^4^Department of Neurology, University Medical Center Hamburg-Eppendorf, Hamburg, Germany; ^5^Department of Clinical Psychology and Psychotherapy, Institute of Psychology–Christian-Albrechts-University, Kiel, Germany

**Keywords:** multiple sclerosis, driving, cognition, driving simulator, driving ability, fatigue, depression, cognitive impairment

## Abstract

**Background and objectives:**

Research on driving ability in people with multiple sclerosis (MS) suggests that they might be at risk for unsafe driving due to MS-related motor, visual, and cognitive impairment. Our first aim was to investigate differences in driving ability and performance between people with MS (PwMS) and those without any neurologic or psychiatric disease (“controls”). Secondly, we determined disease-related factors influencing driving ability in PwMS.

**Methods:**

We prospectively compared standardized performance in a driving simulator between 97 persons with early MS [mean (SD) = 6.4 (7.3) years since diagnosis, mean (SD) Expanded Disability Status Scale (EDSS) = 2.5 (1.4)] and 94 group-matched controls. Participants completed an extensive examination comprising questionnaires and assessments regarding driving, cognitive and psychological factors, as well as demographic and disease-related measures. Between-group comparisons of driving-relevant neuropsychological tests and driving performance were done. Correlations were performed to define demographic and disease-related factors on driving performance in MS.

**Results:**

In a driving simulator setting, PwMS had more driving accidents [T(188) = 2.762, *p* = 0.006], reacted slower to hazardous events [T(188) = 2.561, *p* = 0.011], made more driving errors [T(188) = 2.883, *p* = 0.004] and had a worse Driving Safety Score (DSS) [T(188) = 3.058, *p* = 0.003] than controls. The only disease-related measure to be associated with most driving outcomes was the Wechsler Block-Tapping test (WMS-R) backward: number of accidents (*r* = 0.28, *p* = 0.01), number of driving errors (*r* = 0.23, *p* = 0.05) and DSS (*r* = −0.23, *p* = 0.05).

**Conclusion:**

Driving performance in a simulator seems to be reduced in PwMS at an early stage of disease compared to controls, as a result of increased erroneous driving, reduced reaction time and higher accident rate. MS-related impairment in mobility, vision, cognition, and in psychological and demographic aspects showed no or only minimal association to driving ability, but impairment in different areas of cognition such as spatial short-term memory, working memory and selective attention correlated with the number of accidents, and might indicate a higher risk for driving errors and worse performance. These results show that driving ability is a complex skill with involvement of many different domains, which need further research.

## 1 Introduction

Multiple sclerosis (MS) is a chronic neurodegenerative disease and one of the most common neurological diseases in young adults (1). MS frequently leads to severe disabilities in varying areas such as vision, mobility in upper and lower limbs, and cognition (2–4).

Even at an early stage of disease people with MS (PwMS) show impairments across domains (5). PwMS demonstrate significant differences in most sub-domains of cognition within the first five years of diagnosis when compared to controls (5), with up to 61% of PwMS showing some form of cognitive decline within the first ten years of diagnosis (6). Additionally, there has been evidence of significant physical impairment in early MS, especially walking impairment (5). Walking disabilities have been linked to the worsening ability to perform complex activities of daily living such as driving (7) while simultaneously increasing patients’ dependency on driving in their daily lives (8).

Driving, however, is a complex task, involving many cognitive, visual, and motor domains and impairment in those domains may affect driving ability and driving safety (9, 10). Research shows that 6 to 38% of PwMS fail an on-road driving assessment (11–21). PwMS also make more mistakes while driving (22), are more likely to be involved in an automobile accident, and are 3.4 times more likely to have visits to emergency departments because of automobile accidents than controls (23). However, most PwMS are not aware that they may have deficits in driving at all (24).

Cognitive impairment, which can occur in the early stages of MS (2, 6), may play a role in deciding whether PwMS should cease driving or not. The research on the link between cognition and driving in MS, however, is inconclusive. Most studies indicate a significant relationship (11, 12, 16–18, 21, 25–31), with cognitive impairment in more than one area, or in combination with visual impairment predicting driving ability (11–13, 17, 18, 21, 26). However, some studies have reported that cognitive impairment does not have an impact on driving (32, 33). The impact of comorbidities, such as depression and fatigue in PwMS, has rarely been studied (34). Altogether, a clear understanding of differences in driving ability in MS compared to those without MS is still lacking. Additionally, little is known about whether and how PwMS with and without impairment in domains such as cognition, mobility, and fatigue differ from each other in driving ability.

Little is known regarding problems with specific driving parameters in MS, such as control of speed, tracking stability, recognition of dangerous situations (35). Standardized measures to assess driving ability and standardized evaluation thresholds do not exist or vary across countries, and are largely not evidence-based (36). Additionally, real-world on-road driving assessments can potentially be unsafe for PwMS, assessors, and other road users (32), and do not allow for the creation of standardized reproducible driving settings for group comparisons. It may also be difficult and expensive to evaluate performance details in driving evaluations without modified vehicles (33), especially to assess challenges with a high risk for crashes (37). Therefore, driving simulators present an option to safely and continuously monitor driving performance in PwMS over standardized routes (33).

The aim of this study was to (1) compare driving ability and driving performance of PwMS and controls in a standardized driving simulation, and (2) evaluate the impact of impairment in mobility, vision, cognition, and psychological factors on driving ability in PwMS.

## 2 Methods and materials

### 2.1 Participants and procedure

PwMS were recruited from the MS outpatient clinic in the University Medical Center Hamburg-Eppendorf and through advertisements in our MS-specific newsletter. Control participants were recruited through advertisements (i.e., the hospital newsletter for all employees, hospital Instagram page, and the local newspaper). All participants had to be over 17 years old, have a valid driver’s license and driving experience in the last 6 months. Additionally, the MS group needed to have a confirmed MS diagnoses based on McDonald criteria (38). All MS-subtypes were included. There was no criteria relating to time since diagnosis for PwMS. Participants were excluded if they had a history of other neurological disorders, severe psychiatric disorders (e.g., schizophrenia) and severe simulator sickness (e.g., vomiting or severe vertigo).

Participants were recruited between September 2019 and September 2022, completed a comprehensive examination (see Measures) and the evaluation in the driving simulator. All participants received the study information, and written informed consent was obtained. Participants were asked to complete the questionnaires online, and neuropsychological and visual testing was conducted on site. Driving was assessed in a driving simulator. Overall, assessment time was app. 3 h.

### 2.2 Measures

MS-related patient data (i.e., MS type, disease duration, medication, Expanded Disability Status Scale (EDSS)) were obtained from our clinical database. Beck Depression Inventory (BDI) (39) with recommended cut-offs was used to assess depression. The Fatigue Scale for Motor and Cognitive Functions (FSMC) was used to assess fatigue (40). PwMS additionally completed a questionnaire to assess other features (e.g., medication, MS subtype, quality of life, mobility). Visual acuity was measured via an eye chart. We used Early Treatment Diabetic Retinopathy charts (ETDRS charts) to estimate high contrast visual acuity (in decimal format) at 5 meters (41). Each eye was tested separately, and the use of visual aid (e.g., glasses, contact lenses) was allowed.

Before the driving assessment, participants undertook the following neuropsychological examinations:

1. Brief International Cognitive Assessment for Multiple Sclerosis (BICAMS) (42). The BICAMS comprises: (1) the oral version Symbol Digit Modalities Test (SDMT) (43) to measure information processing speed, (2) the immediate recall subtest of the Brief Visuospatial Memory Test-Revised (BVMT-R) (44) to assess visuospatial learning, and (3) the German “Verbal learning and memory test” (Verbaler Lern- und Merkfähigkeitstest, VLMT) (45) to evaluate the short-term memory (supraspan) and verbal learning.
2. Trail Making Test (TMT) Part A and B (46). Part A evaluates visuo-motor function and visual processing speed whereas part B assesses working memory, cognitive flexibility, executive functioning and visual–spatial capabilities.
3. Wechsler Block-Tapping test (WMS-R block span) (47) forward (fw) and backward (bw) was used to measure the spatial short-term (fw) and working memory (bw).
4. The German “Test battery for attentiveness testing for Mobility” (Testbatterie zur Aufmerksamkeitsprüfung, TAP-M, vers. 2.1./2007) (48) to assess attentional capabilities. Four driving-relevant subdomains were evaluated: (1) alertness (median of reaction time (RT) in milliseconds (ms)), (2) divided attention (median of RT to auditory signals, median of RT to visual signals, number of missed signals, number of errors), (3) visual scanning (median of RT to critical stimuli [a target symbol (a square open at the top) slightly differing from the other symbols (squares with openings either on one of the sides or at the bottom) displayed], median of RT to non-critical stimuli [only non-target symbols displayed], number of missed critical stimuli and number of errors), (4) Go-No-Go to assess selective attention (median of RT, number of missed signals and number of errors).

### 2.3 Driving simulator setting

**Figure 1:**
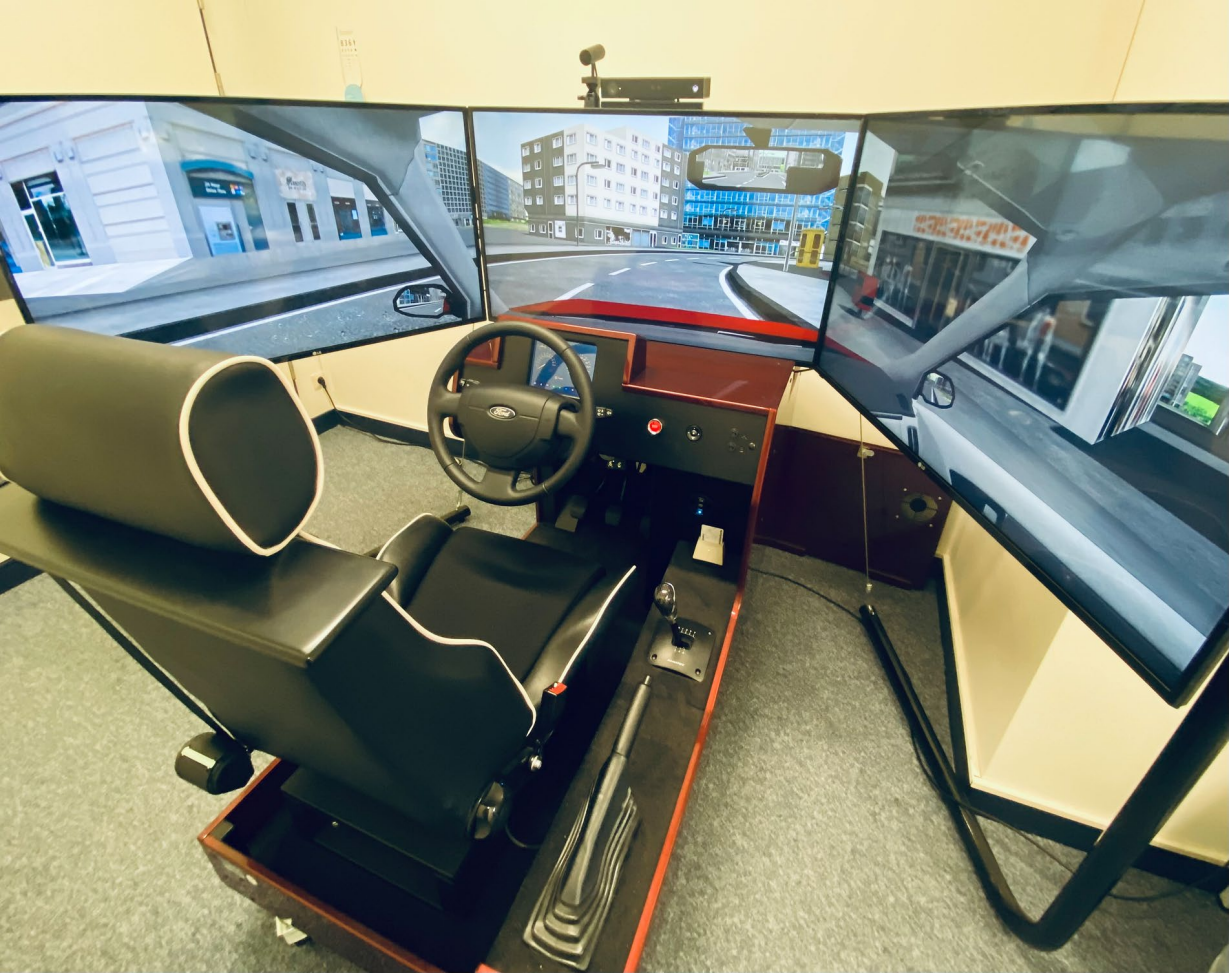
The driving simulator Foerst Model F10-P that was used in our study is shown here.

We used the driving simulator Foerst Model F10-P (Dr.-Ing. Reiner Foerst GmbH, Wiehl; Germany). The driving simulator provides a realistic driver’s cabin of a recreated Ford Fiesta (see Figure 1), with a steering wheel, pedals (brake, accelerator, clutch) and a shift stick. The simulator could be driven either on automatic or manually. A wide screen (2,95 m X 0,55 m, divided into 3 parts) projected the driving scenarios. The screens additionally showed the windscreen, the rear-view mirror and the side mirrors, so the traffic behind could be observed. The dashboard contained a speedometer showing the velocity in km/h; windshield wiper, lights and indicator could be activated manually. An integrated sound system simulated motor, velocity and collision sounds and was used to give directions to the participants. A camera recorded movements of the head as well as the participants’ line of gaze.

The two driving scenarios (training route and main route) consisted of a drive on a rural and an urban road during daylight with good weather conditions. The routes had different hazards implemented, which forced participants to hit the brakes as fast as possible to avoid crashing:

- A car pulling out unexpectedly
- A person running onto the street
- A deer running onto the street
- A parked car on the right side has its door opened unexpectedly.

Depending on individual speed, the training route took approximately 3–6 min, and the main route took 8–13 min.

The following parameters were evaluated to assess participants’ individual driving performance:

1. Number of accidents
2. Mean reaction time (mRT) in seconds (s) to a hazardous event on the road. The sum of all RTs was divided by the number of registered RTs.
3. Driving errors: Thirteen pre-defined typical driving errors were calculated (see Table 1) (49). The driving errors were detected and recorded automatically (log file) by the simulator. The driving errors were used to determine the Number of Driving Errors (NDE) and the Driving Safety Score (DSS). The NDE is the sum of all driving errors. The DSS is a composite score considering both the frequency and the seriousness of driving errors. The seriousness of the driving errors are scored with a severity factor weighted according to safety-clinical relevance with errors like not using a turn signal and driving a little slow rated as less severe, and errors like missing a red light or getting into an accident rated as severe.

**Table 1:** Definition and severity factor of driving errors.

Driving error category	Definition	Severity factor
No response to an event – accident	Accident without applying the brakes before	8
Accident	Accident with delayed activation of the brakes	7
Endangering other road users	Situation in which a person, animal or object can be harmed	6
Ignoring red light	Ignoring a red light on traffic lights	6
Wrong side of the road	All 4 wheels are driving on the other / opposing side of the road	4
Leaving roadway	At least one wheel is crossing the road marking	3
Knocking over marker posts	Collision with a marker post	3
Driving too fast	Exceeding the permitted speed limit with >10 km/h	2
Driving a little slowly	Duration of whole ride longer than one standard deviation (SD) compared to the groups’ means	1*
Driving way too slowly	Duration of whole ride longer than two SD compared to the groups’ means	2*
Missing indicator and shoulder check	Activating the indicator and shoulder check are not conducted correctly	2
Missing indicator when making a lane change	Indicator activation is not conducted correctly when making a lane change	1
Missing shoulder check	Shoulder check has to be conducted with a head rotation of at least 20 degrees to the left or right side	1

Different driving errors with their definition and severity factor according to safety-clinical relevance. These seriousness-weighted driving errors are used to calculate the Driving Safety Score (DSS). *These two driving error categories were later combined for statistical analysis. Adapted from Fründt et al. (49).

### 2.4 Statistical analysis

The driving data were recorded in a simulator-provided log file and transformed into a SPSS file. The results of demographic and MS-specific data, questionnaires/scores and neuropsychiatric tests were added afterwards. IBM SPSS version 29 was used for all statistical analysis.

Descriptive analyses of demographic and clinical data were performed. Quantitative variables are described by their mean (M) and standard deviation (SD). Qualitative variables are expressed in percentages. Group comparisons (between PwMS and controls) were performed using t-tests of means and Welch-test, chi-square tests (χ^2^) were used to analyze qualitative variables, and to compare quantitative variables. Effect sizes (ES) were calculated with Cohen’s d.

Correlations were performed using Pearson’s (for linear variables) and Spearman’s (for ordered variables) correlation coefficient to investigate associations between all measures (i.e., demographic data, MS-specific data, results of the neuropsychiatric and neuropsychological tests) and driving performance.

To identify differences in PwMS, exploratory extreme value analyses were performed. For the extreme values, we used the lowest and the highest quartile of data (age, EDSS) in the group of PwMS and conducted t-tests for independent measures. To identify differences between the groups related to fatigue vs. non-fatigue and moderate/severe depression vs. non-depression, we used the appropriate cut-offs.

We conducted one-way ANCOVAs with the different driving outcomes (mRT, number of accidents, NDE or DSS) as the dependent variable, group (PwMS vs. controls) as the independent variable and depression, fatigue and visual acuity as covariates, since we found significant group differences regarding these outcomes.

A significance value of *α* = 5% (*p* < 0.05) was used in all analyses.

### 2.5 Ethical statement

The study was approved by the local ethics committee and is in accordance with the Declaration of Helsinki (Votum No. 11/2019-PTK-HH).

## 3 Results

A total of 247 people were recruited (121 PwMS and 126 age-and gender-matched controls). 97 PwMS and 94 controls completed the main route driving scenario. 23 PwMS and 32 controls were excluded from the analysis because they could not finish the driving scenario due to motion sickness, two participants (one PwMS and one control participant) were excluded for not having a valid driving license at the time of testing.

### 3.1 Group differences in clinical scores, neuropsychological, and psychological measures

PwMS (39 ± 11.07 years, 51% women, 49% men, 69% relapsing remitting MS (RRMS)) and controls (37 ± 15.27 years, 60% women, 40% men) did not differ on any demographic data (Table 2). Both groups differed in visual acuity [T(181) = −11.86, *p* < 0.001], with PwMS having poorer eyesight than controls.

**Table 2:** Demographic and clinical data of PwMS and controls.

Clinical parameters/scores	PwMS *n* = 97 Mean (SD) [Min-Max]	Controls *n* = 93 Mean (SD) [Min-Max]	*p*-value
Age (years)	38.96 (11.1)	37.43 (15.3)	0.432^a^
Gender (%)			0.179^b^
Women	49 (50.5%)	56 (59.6%)	
Men	48 (49.5%)	37 (40.4%)	
Other	0	0	
Education			0.065^b^
≤ 10 years	30 (30.9%)	14 (15.1%)	
≤ 13 years	31 (32.0%)	35 (37.6%)	
University degree	35 (36.1%)	44 (47.3%)	
Employment			0.121^b^
Full-time	48 (49.5%)	48 (51.6%)	
Part-time	19 (19.6%)	20 (21.5%)	
In education/training	5 (5.2%)	17 (18.3%)	
Unemployed	5 (5.2%)	2 (2.2%)	
Pension (e.g., old age, disability)	6 (6.2%)	4 (4.3%)	
Other (e.g., homemaker)	3 (3.1%)	1 (1.1%)	
Driving experience (years)	19.85 (10.99) [1–52]^y^	18.98 (15.27) [1–57]^z^	0.667^a^
Weekly driving (days a week)	3.63 (1.88) [1-7]^A^	3.03 (1.96) [1-7]^B^	0.064^a^
Routes driven in a regular week			0.424^b^
Familiar routes (80% familiar)	65 (67.0%)	58 (62.4%)	
Mostly familiar (60% familiar)	14 (14.4%)	19 (20.4%)	
Mostly unfamiliar (40% familiar)	3 (3.1%)	7 (7.5%)	
Unfamiliar routes (20% familiar)	5 (5.2%)	6 (6.5%)	
No information available	10 (10.3%)	3 (3.2%)	
MS subtype (%)			
RRMS	67 (69.1%)		
SPMS	6 (6.2%)		
PPMS	14 (14.4%)		
Unclear	10 (10.3%)	-	**-**
Disease duration	6.14 (7.32) [0-23]^x^	-	-
EDSS Mean (SD)	2.48 (1.40)		
EDSS Median	2.5		
Visual acuity	90.5 (19.0)	102.36 (17.72)	<0.001^a^

Demographic data of PwMS and controls are given as mean ± standard deviation and minimum and maximum, number of patients (*n*) and percentage (%). There were no statistically significant group differences. ^a^Result of Welch-Test. ^b^Result of Pearson chi-squared Test. ^x^N = 94 ^y^N = 86 ^z^N = 87 ^A^N = 70 ^B^N = 72 ^C^N = 89. PwMS, people with multiple sclerosis; SD, Standard deviation; MS, multiple sclerosis; RRMS, Relapsing Remitting Multiple Sclerosis; SPMS, Secondary Progressive Multiple Sclerosis; PPMS, Primary Progressive Multiple Sclerosis; EDSS, Expanded Disability Status Scale.

The results of the neuropsychological and psychological assessment are reported in Table 3. The groups differed on TAP-M Alertness; PwMS had slower reaction times (RT) [T(169.25) = 5.617, *p* < 0.001] than controls. For TAP-M Visual Scanning, the groups showed differences for their RTs to critical stimuli [T(187) = 3.651, *p* < 0.001] and non-critical stimuli [T(187) = 2.705, *p* = 0.007], with PwMS having reduced RTs compared to controls. For the TAP-M Go-No-go, testing selective attention, PwMS had reduced RT [T(178.84) = 5.297, *p* < 0.001] than controls. For TAP-M divided attention, PwMS and controls only differed in their RT for visual stimuli, with PwMS reaction slower than controls [T(177.31) = 2.773, *p* = 0.006]. There were no significant differences for the RT to auditory stimuli on TAP-M divided attention or the number of misses or errors in any of the TAP-M tests.

**Table 3:** Results of neuropsychological and psychological tests.

Neuropsychological test		PwMS (*n* = 97) Mean (SD)	Controls (*n* = 93) Mean (SD)	Statistics (*p-*value)	ES (d)
SDMT		55.9 (11.6)	60.7 (10.7)	**0.004** ^**a**^	−0.42
BVMT Learning (1–3)		25.3 (6.7)	28.6 (5.9)	**<0.001** ^**a**^	−0.52
VLMT	Supraspan	7.8 (2.1)	8.8 (1.8)	**0.001** ^**a**^	−0.49
	Verbal learning (1–5)	56.0 (10.3)	61.2 (7.4)	**<0.001** ^**b**^	−0.55
TMT part A (in s)		24.7 (12.6)	22.4 (6.8)	0.135^a^	0.23
TMT part B (in s)		57.8 (22.7)	53.2 (19.0)	0.145^a^	0.22
WMS-R block span forwards		9.1 (2.0)	9.3 (1.8)	0.353^a^	−0.14
WMS-R block span backwards		8.5 (1.59)	8.6 (1.6)	0.585^a^	−0.08
Alertness	RT (ms)	249.0 (42.2)	219.7 (28.5)	**<0.001** ^**b**^	0.75
Visual Scanning	RT – crit. (ms)	2916.3 (729.9)	2554.1 (628.3)	**<0.001** ^**a**^	0.51
	RT – not crit. (ms)	5412.6 (1233.8)	4911.8 (1310.6)	**0.007** ^**a**^	0.39
	Missed crit. Signals	7.8 (6.1)	7.8 (6.6)	0.958^a^	0.01
	Errors	0.2 (0.4)	0.2 (0.4)	0.938^a^	0.02
Selective attention (Go-NoGo)	RT (ms)	429.9 (71.9)	381.0 (54.6)	**<0.001** ^**b**^	0.71
	Missed signals	0.2 (0.6)	0.1 (0.8)	0.652^a^	0.07
	Error	0.8 (1.0)	1.0 (1.4)	0.126^a^	−0.22
Divided attention	RT – audit. (ms)	598.7 (93.6)	579.3 (164.5)	0.315^a^	0.15
	RT – visual (ms)	788.9 (120.0)	744.7 (93.1)	**0.006** ^**b**^	0.39
	Missed signals	1.6 (1.7)	1.5 (2.4)	0.682^a^	0.06
	Errors	0.9 (1.2)	1.3 (2.4)	0.148^b^	0.21
Depression (BDI score)		12.26 (10.4)	5.64 (5.77)	**<0.001** ^**b**^	0.74
No depression		41 (42.3%)	70 (75.3%)		
Minimal depression		16 (16.5%)	11 (11.8%)		
Mild depression		12 (12.4%)	5 (5.4%)		
Moderate depression		7 (7.2)	3 (3.2%)		
Severe depression		11 (11.3%)	1 (1.1%)		
Fatigue (FSMC score)		53.76 (18.22)	37.92 (12.49)	**<0.001** ^**b**^	0.91
No fatigue		23 (24%)	61 (66%)		
Mild fatigue		16 (16%)	18 (19%)		
Moderate fatigue		19 (20%)	9 (10%)		
Severe fatigue		29 (30%)	2 (2%)		

Results of PwMS and controls are given as mean ± standard deviation and minimum and maximum. Statistically significant results are in bold typeface. ^a^Calculated with *t*-tests. ^b^Calculated with Welch-Test. PwMS, people with multiple sclerosis. SD, Standard deviation; ES, Effect size; SDMT, Symbol Digit Modalities Test; BVMT, Brief Visuospatial Memory Test-Revised; VLMT, Verbal learning and memory test; TMT, Trail Making Test; WMS-R, Wechsler Block-Tapping test; TAP-M, Test battery for attentiveness testing, version mobility; RT, Reaction time; ms, milliseconds; Crit., critical; Audit., auditory; BDI, Beck Depression Inventory; FSMC, The Fatigue Scale for Motor and Cognitive Functions.

PwMS and controls also differed on SDMT [T(186) = −2.909, *p* = 0.004], BVMT [T(188) = −3.702, *p* < 0.001], VLMT supraspan [T(185) = −3.483, *p* = 0.001], and VLMT verbal learning [T(173,60) = −3.967, *p* < 0.001], with PwMS performing worse than controls. We did not find a significant between-group difference in TMT-A, TMT-B, WMS-R block span forwards, and WMS-R block span backwards (all *p* > 0.05).

41 PwMS (42%) and 70 controls (75%) did not show signs of depression according to BDI-II cut-off scores. Overall, PwMS were more depressed than controls [T(133.41) = 6.620, *p* > 0.001]. 61 controls (66%) and 23 PwMS (24%) did not show any signs of fatigue. 29 (30%) PwMS were classified as having severe fatigue while only 2 (2%) controls reported severe fatigue. PwMS scored higher on the FSMC [T(151.63) = 15.836, *p* > 0.001] measuring fatigue, indicating higher levels of persistent tiredness and exhaustion in the MS-group than in controls.

### 3.2 Driving simulator performance

The results of the main driving parameters (i.e., mRT, number of accidents, DSS and NDE) are given in Table 4. PwMS reacted slower to hazardous events (mRT) [T(188) = 2.561, *p* = 0.011], had more accidents [T(188) = 2.762, *p* = 0.006], higher NDE [T(188) = 2.883, *p* = 0.004], and higher DSS [T(188) = 3.058, *p* = 0.003] than controls.

**Table 4:** Driving performance results.

Driving performance parameters	PwMS Mean (SD)	Controls Mean (SD)	Statistics (*p*-value)	ES
Driving time (DT) in s	554 (77)	541 (64)	0.226	0.18
Mean Reaction time (mRT) in s	1.61 (0.18)	1.54 (0.17)	**0.011**	0.37
Number of Accidents	1.37 (1.18)	0.92 (1.03)	**0.006**	0.40
No. of Driving Errors (NDE)	4.25 (3.05)	3.11 (2.33)	**0.004**	0.41
Driving Safety Score (DSS)	18.82 (13.15)	13.60 (10.13)	**0.003**	0.43

Driving simulator results of PwMS and controls are given as mean ± standard deviation and minimum and maximum. Significant results are bolted. PwMS, people with multiple sclerosis; SD, Standard deviation; ES, Effect size.

Since we found differences between the groups on visual acuity (see Table 2), depression and fatigue (see Table 3), we conducted one-way ANCOVAs to determine the difference between PwMS and controls on the driving outcomes, controlling for visual acuity, depression and fatigue.

After adjusting for depression, the interaction between the groups (PwMS vs. controls) and the driving outcomes remained significant, indicating that depression had no significant interaction in the group differences. PwMS still showed slower mRT [*F*(1, 174) = 6.16, *p* = 0.014, η^2^ = 0.034], had more accidents [*F*(1, 174) = 8.557, *p* = 0.004, η^2^ = 0.047], higher NDE [*F*(1, 174) = 6.758, *p* = 0.010, η^2^ = 0.038] and higher DSS [*F*(1, 174) = 9.427, *p* = 0.002, η^2^ = 0.051] than controls.

Similarly, the interaction between both groups remained significant when controlled for fatigue. PwMS reacted slower [*F*(1, 174) = 5.22, *p* = 0.024, η^2^ = 0.029], made more accidents [*F*(1, 174) = 6.298, *p* = 0.013, η^2^ = 0.035], had higher NDE [*F*(1, 174) = 7.724, *p* = 0.006, η^2^ = 0.043] and higher DSS [*F*(1, 174) = 6.470, *p* = 0.012, η^2^ = 0.036] than controls.

Adjusting for visual acuity, the interaction between PwMS and controls remained significant for the number of accidents [*F*(1, 180) = 9.802, *p* = 0.002, η^2^ = 0.052], NDE [*F*(1, 180) = 13.753, *p* = 0.000, η^2^ = 0.071] and DSS [*F*(1, 180) = 13.096, *p* = 0.000, η^2^ = 0.068]. No significant interaction was found for mRT [*F*(1, 180) = 3.083, *p* = 0.081, η^2^ = 0.017] when adjusted for visual acuity, showing that visual acuity has a significant interaction in the group difference for mRT.

### 3.3 Correlations between all measures and driving outcomes in PwMS

To explore whether cognitive impairment in MS relates to driving ability, we computed correlations between the neuropsychological measures and the driving outcomes (Table 5).

**Table 5:** Correlations with driving-related outcomes (PwMS).

Parameters		Mean RT (s)	No. of accidents	NDE	DSS
Age		0.03	−0.18	−0.05	−0.02
Sex		0.13^a^	−0.04^a^	−0.09^a^	−0.04^a^
Time since diagnosis (years)		−0.07	−0.11	−0.08	−0.01
EDSS		0.11	0.05	0.01	0.20
Visual acuity		−0.14	0.09	0.21*	0.14
Driving experience (years)		0.04	−0.21*	−0.06	−0.05
Weekly driving (days a week)		−0.13	−0.19	−0.05	−0.07
Familiarity of routes driven in a regular week		−0.24* ^**a**^	−0.13^a^	0.07^a^	−0.11^a^
SDMT		0.00	−0.09	0.06	−0.02
BVMT Learning (1–3)		0.03	−0.21*	0.04	−0.02
VLMT	Recall (1–5)	−0.13	−0.12	0.01	−0.08
	Supraspan	−0.11	−0.05	0.05	−0.06
TMT part A		−0.01	−0.08	−0.05	0.02
TMT part B		−0.03	−0.07	−0.08	−0.03
WMS-R block span forwards		−0.10	0.22*	0.19	0.18
WMS-R block span backwards		−0.03	0.28**	0.23*	0.23*
Alertness	RT (ms)	0.17	−0.06	0.02	0.03
Visual Scanning	RT – crit. (ms)	0.12	−0.09	−0.05	−0.03
	RT – not crit. (ms)	0.14	−0.09	−0.07	−0.05
	Missed crit. Signals	0.02	−0.05	0.01	0.05
	Errors	−0.21*	−0.04	0.07	−0.00
Selective attention	RT (ms)	0.08	−0.18	−0.15	−0.08
	Missed signals	0.10	0.25*	0.11	0.17
	Errors	0.16	0.12	0.02	0.06
Divided attention	RT – auditory (ms)	0.13	−0.03	−0.05	−0.02
	RT – visual (ms)	0.05	−0.18	−0.11	−0.06
	Missed signals	−0.04	−0.15	−0.10	−0.05
	Errors	0.03	−0.05	−0.03	−0.01
Depression		0.01	−0.06	−0.05	−0.06
Fatigue		−0.01	−0.05	−0.07	−0.01

Correlations (Pearson or Spearman^(a)^) of the Driving Safety Score (DSS) and the number of accidents with clinical and neuropsychological data of PwMS (*n* = 97) are shown with *r*-values and *p*-values. Significant correlations are marked bold, *p = 0.05, **p = 0.01, ***p = 0.001. PwMS, people with multiple sclerosis; RT, reaction time; s, seconds; No., number; NDE, No. of Driving Errors; DSS, Driving Safety Score; EDSS, Expanded Disability Status Scale; SDMT, Symbol Digit Modalities Test; BVMT, Brief Visuospatial Memory Test-Revised; VLMT, Verbal learning and memory test; TMT, Trail Making Test; WMS-R, Wechsler Block-Tapping test; TAP-M, Test battery for attentiveness testing, version mobility; RT, Reaction time; ms, milliseconds; Crit., critical.

A negative correlation was shown between mRT and the number of errors in TAP-M Visual scanning (*r* = −0.21, *p* = 0.05) and mRT and the familiarity of routes driven in a regular week (*r* = −0.24, *p* = 0.026), indicating PwMS who regularly drove more unfamiliar routes reacted quicker to hazardous events than PwMS who drove unfamiliar routes less often.

Significant positive correlations were found between the number of accidents and missing the stimuli in TAP-M Go-No-go (*r* = 0.254, *p* = 0.012), Wechsler Block-Tapping test (WMS-R) block span forwards (*r* = 0.225, *p* = 0.031), WMS-R block span backwards (*r* = 0.281, *p* = 0.007) and the mRT to hazardous events (mRT) (*r* = 0.277, *p* = 0.006). Significant negative correlations were found between the number of accidents and driving experience in years (*r* = −0.213, *p* = 0.049) and BVMT (*r* = −0.208, *p* = 0.041), indicating that PwMS who have more accidents have less driving experience, remember less in BVMT.

The NDE correlated with visual acuity (*r* = 0.21, *p* = 0.05) and WSM-R blockspan backward (*r* = 0.23, *p* = 0.05). For DSS, significant positive correlations were found only with the WMS-R block span backwards (*r* = 0.234, *p* = 0.025).

No correlation remained significant when controlled for multiple comparisons.

### 3.4 Additional analysis

An exploratory analysis of the data for the extreme groups in reaction time, number of accidents, NDE and DSS was conducted. We found no significant differences in driving outcomes for age, EDSS, cognitive impairment and depression.

Results of the correlations between all measures (i.e., demographic, cognitive and psychological measures) and the driving outcomes in controls can be found in the Supplementary material. The only significant correlations found for controls were between the number of accidents and driving experience in years (*r* = −0.228, *p* = 0.033), between mRT and the number of errors in TAP-M Go-No-go (*r* = 0.210, *p* = 0.043), and mRT and the number of errors in TAP-M divided attention (*r* = −0.241, *p* = 0.020).

## 4 Discussion

This is one of the few studies that investigated differences in driving ability between PwMS and controls in a standardized driving simulation, and evaluated the impact of impairment in different functional areas such as mobility, vision, cognition and psychological factors on driving performance in PwMS. To our knowledge, this is the largest study of PwMS assessed in a driving simulator addressing these specific associations.

### 4.1 Group comparisons (PwMS vs. controls)

PwMS performed worse in a driving simulator compared to controls. This supports previous findings assessing driving in PwMS (27–29, 32, 37). PwMS reacted slower to hazardous events, had more accidents, made more driving errors (NDE), and had higher severe errors (DSS) than controls, indicating poorer driving ability within the simulator setting.

Most accidents occurred when the participants were driving fast (on a country road without speed limits) or when they faced sudden critical challenges (e.g., a child obscured by a truck suddenly stepping onto the street or a car door opening unexpectedly). This suggests that delayed reaction time resulting in late braking is the main cause of accidents in MS patients. Another study found the increased accident rate in PwMS particularly in monotonous driving situations and attributed this to a lower arousal rate compared to controls (28). However, in our study, accidents occurred in both monotonous and non-monotonous situations.

PwMS also made more NDE and had more DSS than controls, which shows that the slower reaction time alone may not be the only reason for the higher number of accidents in PwMS. In other studies assessing driving performance in a simulated setting, PwMS were also shown to have performed worse in different areas of driving, such as the standard deviation of lateral position (27, 29), adjustments to stimuli errors (32, 37), greater variability in speed maintenance (29), and a greater number of concentration faults (28). While those errors do not completely translate to the scores used in this study, aspects of all of them can be found in the NDE and the DSS, with speed, lateral position and stimuli errors being taken into account in both scores. The use of different driving simulators with different settings, scores and driving performance measures make it difficult to compare others’ findings with ours, but they indicate (almost consistently across studies) that driving performances is impaired in PwMS.

Both groups did not differ on demographic parameters (e.g., age, sex, employment, etc.). However, differences were found in visual acuity, in neuropsychological scores (e.g., subtests of the TAP-M, SDMT, BVMT, VLMT) and psychological measures (depression and fatigue), which is understandable, considering MS can have an impact on those areas (2, 3, 8). MS-specific impairment seems to have an impact on driving ability in PwMS, however, as shown previously, the extent of the impact remains unclear and somewhat inconsistent (34).

### 4.2 Impact of demographic and clinical factors on driving ability

In prior research, different MS-related impairments in terms of mobility, vision, cognition, and in psychological and demographic aspects were found to negatively impact driving ability, but the data were inconsistent (34, 35).

In our study, no demographic features were associated with driving outcomes, except for driving experience. Driving experience was related to the number of accidents – PwMS with less driving experience had more accidents than those with more experience. The same association was found for controls, and was the only significant correlation for both PwMS and controls. Prior research showed that drivers with 6–10-year driving experience had the most number of accidents, followed by drivers with 3-year, while drivers with 20+ years driving experience account for the lowest number of accidents (50). This demonstrates that this is also perhaps a complex (non-linear) association, with more accidents happening initially due to lack of experience in dealing with hazardous events, and a subsequent increase in accidents with increased confidence in driving but not with associated skills, followed by a reduction with improved confidence and skills.

No significant relations were found between EDSS and any of the driving outcomes, which is in line with previous research on driving ability in PwMS, demonstrating that (mild) physical disability has no or only partial impact on driving performance (34, 35).

Visual acuity was related only to NDE, with PwMS with higher visual acuity making more mistakes than PwMS with less visual acuity. This is especially interesting, as multiple studies have found lower visual acuity to have a significant impact on driving ability in PwMS (14, 16, 26). A possible reason for this result could be that in our study we only checked for farsightedness due to social distancing rules during the COVID-19 pandemic. Checking nearsightedness and other visual functions (e.g., contrast sensitivity, glare recovery, depth perception) would have been important, especially considering that watching a monitor (on which the driving simulation was shown) requires good nearsightedness and contrast sensitivity. It is possible that participants with good farsightedness are more likely to have problems with nearsightedness, which would explain the positive relation to NDE. A main effect was found for visual acuity and mRT, indicating that PwMS reacting slower to hazardous events may be a result of worse visual acuity. PwMS differed significantly in visual acuity from controls, so a reason for their slower reaction time could be that they did not immediately see the hazardous event. No main effect or interaction was found between visual acuity and the other driving outcomes.

Our MS group showed clinically relevant and increased fatigue compared to controls. However, we did not find fatigue to have an impact on driving performance, which is in line with prior research (12, 25–27). Additionally, no differences were found between PwMS and controls in the driving outcomes, when we controlled for fatigue. A possible reason for these results could be the brevity of the driving assessment (about 9 min). Longer driving sessions may reveal differences between PwMS with and without fatigue better. On the other hand, a study, which had PwMS drive on a standardized road for 45 min, found that fatigue had no impact on driving performance (12). Therefore, perhaps PwMS have learnt to compensate or deal with their fatigue while driving.

Depression was not related to any of the driving outcomes. While some research has suggested that driving performance may be affected in individuals with depression (51), others have found inconclusive evidence of this association in PwMS (34). Since PwMS and controls significantly differed in depression, we investigated the group differences in the driving outcomes and controlled them for depression. No main effect or interaction was found in this study, indicating that while PwMS were more depressed than controls, differences in driving ability were not a result of depression. Prior research has also shown that PwMS who have symptoms of depression tend to have higher levels of cognitive impairment (52, 53). In our study, cognitive impairment was also greater in PwMS in some of the neuropsychological tests than in controls.

Whether cognitive impairment relates to driving ability is unclear, because prior research has shown the data to be inconsistent and contradictory (34, 35), with some studies suggesting cognitive impairment can predict driving ability (11–13, 17, 18, 21, 26), while other no impact on driving (32, 33). Studies reporting an impact of cognitive impairment on driving ability have differed in which areas of cognition are important, with contradictory results (34, 35). Similarly, our results on the impact of cognition on driving ability were inconsistent. We did find all driving outcomes (mean reaction time, number of accidents, NDE and DSS) to correlate with at least one neuropsychological measure but no test was consistently associated with all driving outcomes. The only test score that correlated with 3 out of 4 of the driving outcomes was the WMS-R block span. But, it should be noted that no correlation remained significant when controlled for multiple comparisons.

The results of the correlations of the WMS-R block span forward and backward showed that visual–spatial short-term and working memory are associated with having more accidents. The WMS-R block span backward also showed a positive correlation to NDE and DSS, indicating that a better performance in the test means making more driving errors and having more severe driving errors. We would have hypothesized that a good performance in the WMS-R would indicate the opposite: fewer accidents, lower NDE and lower DSS. A possible explanation for these results could be that implicit awareness of good visual–spatial short-term and working memory might make PwMS more confident in their own ability, which in turn might lead to driving less carefully. A prior study showed that PwMS drove more and had more driving incidents (e.g., accidents and traffic violations) if they felt they did not have cognitive deficits for which they needed to compensate (24).

For all other measures that were related to a driving outcome the association was as hypothesized, with worse performance in the test being related to worse driving performance. PwMS who had more accidents remembered less in BVMT and missed more signals in the selective attention test, while PwMS who reacted more slowly to hazardous events made more errors in the visual scanning test.

Overall, many of the neuropsychological measures only showed minimal to no association to driving ability, especially scores frequently used in studies investigating driving ability like TMT-A, TMT-B and SDMT. Generally, prior research on driving performance in other cohorts (such as older drivers or those with Parkinson’s disease) has found cognitive impairment to be a strong predictor for impaired driving for those groups (54). In PwMS, those results were, similar to the results of this study, less convincing (34, 35). A reason for this might be that the PwMS tested in this study were relatively young and had early MS. And, while PwMS did significantly differ from controls in some of the neuropsychological tests in this study, there was no sign of impairment in others (e.g., TMT-A and B, WMS-R fw and bw), indicating cognitive impairment is still mild in this cohort. Prior studies found older PwMS to have more impairment in cognition than young and middle-aged PwMS (55) and that PwMS show a greater decline in physical ability than in cognition in the first 10 years of their disease (56). Therefore, the MS cohort may have been too young and not cognitively impaired enough to show significant differences. Although, it is also possible that the tests used were not sensitive enough to show the differences.

### 4.3 Limitations

While this, to our knowledge, is the largest study to assess cognition, related clinical and psychological factors and driving ability in a simulator, we acknowledge that there are some limitations. Firstly, the driving simulator setting might not exactly reflect real-life driving. For instance, in such a study setting, where people are being closely monitored, they may be extra vigilant or nervous compared to unobserved on-road driving. However, research has shown that driving simulators, like the one we used, closely mimic on-road driving behaviors and reflect on-road driving properly (57).

Secondly, while we tried to explore different cognitive and neuropsychological domains by using different tests, we acknowledge that there is an overlap in domains assessed in each of these tests (58). For instance, while the WMS-R block span purportedly assesses visuospatial memory, it also relies on attention and concentration abilities. Therefore, the precise nature of the effect of each of these cognitive domains should be treated with some caution.

Also, it should be noted, that most of our MS participants, being early on in their MS journey, only had low levels of physical and cognitive disability. It is possible that most of the MS-impairment did not show an association on driving ability because the population was not impaired enough.

### 4.4 Clinical and scientific impact

Our results show that PwMS, even those with early MS, had more accidents, slower reaction time to hazardous events, made more driving errors and had more severe mistakes in a driving simulation than controls, which could indicate that PwMS have more difficulties to drive safely compared to controls. Unfortunately, as of now, there is no validated tool to identify PwMS who might not be able to drive safely without extensive testing because there is a lack of validated predictive test batteries for driving ability (34). As the extent of impact MS-specific impairment has on driving ability remains unclear and inconsistent, further research into the topic is necessary. Multiple studies, including ours, could show a difference in driving ability between PwMS and controls, however, consistent predictors for impaired driving are still lacking (27–29, 32, 37). While cognition may impact driving ability, it is unclear to what extent and in which areas, as we found an association between all driving outcomes with at least one neuropsychological measure, but no test was consistently associated with all driving outcomes. This suggests that it is not one cognitive area alone that impacts driving ability.

Future research might benefit from investigating older PwMS, with longer disease duration and more disease progression (particularly greater cognitive and physical impairment), because this may be useful in exploring how MS-specific impairments worsen driving ability. The results of our study show that PwMS have more problems in driving compared to controls, but it remains unclear which areas of MS-specific impairment impact driving ability in PwMS exactly and how great this impact is. Many of the regularly used neuropsychological and physical tests appear not to be sensitive enough for those with mild impairments, so having PwMS with greater impairments might help in identifying different factors that affect driving ability. It might also be beneficial to have longitudinal studies in this area, to see how disease progression and MS-specific impairment affects driving ability over time. Additionally, it might be advisable to use different tools for assessment of impairment, e.g., The Stroke Driver Screening Assessment (59) (SDSA) and the Useful Field of View test (60, 61) (UFOV), have both been found to be promising tools (34, 35).

It should be noted that while we found differences between PwMS and controls in driving, this does not automatically translate to PwMS not being able to drive safely. There should be further research, especially research with on-road driving tests, as the data we have as of now would not justify recommending driving cessation for all PwMS. However, it shows that this is an important issue that should be discussed with PwMS early on. This is especially important as many physicians do not make recommendations on driving or only do so after an adverse event (e.g., a car crash) has already taken place (61). To ensure PwMS are aware of the possibility of declining driving ability and allow them to compensate for it, physicians should routinely discuss driving issues.

## Data availability statement

The raw data supporting the conclusions of this article will be made available by the authors, without undue reservation.

## Ethics statement

The studies involving humans were approved by Psychotherapeutenkammer Hamburg. The studies were conducted in accordance with the local legislation and institutional requirements. The participants provided their written informed consent to participate in this study.

## Author contributions

SS: Conceptualization, Data curation, Formal analysis, Investigation, Methodology, Project administration, Writing – original draft, Writing – review & editing. RN: Conceptualization, Writing – original draft, Writing – review & editing. CH: Conceptualization, Funding acquisition, Investigation, Methodology, Project administration, Writing – original draft, Writing – review & editing. CB: Conceptualization, Methodology, Writing – original draft, Writing – review & editing. AP: Formal analysis, Methodology, Writing – original draft, Writing – review & editing. JP: Conceptualization, Formal analysis, Funding acquisition, Investigation, Methodology, Project administration, Resources, Supervision, Writing – original draft, Writing – review & editing.
