# Supplementary material for: Factors affecting driving performance in patients with Multiple Sclerosis – still an open question
Source: Front Neurol. 2024 Feb 28;15:1369143. doi: 10.3389/fneur.2024.1369143 (PMC10933050; doi:10.3389/fneur.2024.1369143)
Supplement: Supplementary file 1 [file Table_1.DOCX]

**Supplementary materials**

**Supplement 1.** Table with correlation of the driving-related outcomes for controls.

**Table 1: Correlations with driving-related outcomes (controls)**

| **Parameters** | | | **Mean RT (s)** | **No. of accidents** | **NDE** | **DSS** |
| --- | --- | --- | --- | --- | --- | --- |
| Age | | | 0.15 | -0.20 | -0.07 | -0.07 |
| Sex | | | 0.04^a^ | 0.17^a^ | 0.02^a^ | 0.12^a^ |
| Visual acuity | | | -0.08 | 0.03 | 0.04 | 0.00 |
| Depression | | | 0.02 | -0.08 | -0.11 | -0.07 |
| Fatigue | | | 0.09 | 0.07 | -0.05 | 0.04 |
| Driving experience (years) | | | 0.14 | **-0.23*** | -0.11 | -0.10 |
| Alertness | RT (ms) | | -0.07 | -0.07 | -0.15 | -0.11 |
| Visual Scanning | RT – crit. (ms)  RT – not crit. (ms)  Missed crit. signals  Errors | | 0.13  0.10  0.03  -0.18 | 0.07  0.04  -0.01  0.13 | -0.03  0.03  0.02  0.15 | -0.06  0.07  0.01  0.10 |
| Selective attention | | RT (ms)  Missed signals  Errors | 0.15  -0.18  **0.21*** | 0.01  0.00  0.124 | -0.06  -0.06  0.02 | 0.02  -0.05  0.09 |
| Divided attention | | RT – auditory (ms)  RT – visual (ms)  Missed signals  Errors | 0.02  0.11  0.11  **0.24*** | -0.10  -0.00  0.07  0.11 | -0.06  -0.07  -0.04  -0.09 | -0.03  0.09  0.03  -0.00 |
| SDMT | | | -0.02 | 0.03 | -0.14 | -0.12 |
| TMT part A | | | 0.21 | 0.19 | 0.14 | 0.17 |
| TMT part B | | | 0.09 | 0.14 | 0.19 | 0.21 |
| WMS-R block span forwards | | | -0.10 | 0.12 | 0.195 | 0.12 |
| WMS-R block span backwards | | | -0.17 | 0.01 | -0.06 | -0.08 |
| BVMT Learning (1-3) | | | 0.08 | 0.10 | -0.09 | -0.01 |
| VLMT | | Recall (1-5) Supraspan | -0.07  -0.01 | -0.04  -0.09 | -0.01  -0.08 | -0.05  -0.14 |

*Correlations (Pearson or Spearman^(a)^) of the Driving Safety Score (DSS) and the number of accidents with clinical and neuropsychological data of controls (n=93) are shown with r-values and p-values. Significant correlations are marked bold, *p=0.05, **p=0.01, ***p=0.001. Initialisms: RT = reaction time. s =* seconds*. No. = number. NDE = No. of Driving Errors. DSS = Driving Safety Score. TRT = Reaction time. ms = milliseconds. Crit. = critical. SDMT = Symbol Digit Modalities Test. TMT = Trail Making Test. WMS-R = Wechsler Block-* *Tapping test. BVMT = Brief Visuospatial Memory Test-Revised. VLMT = Verbal learning and memory test.*
